# Effect of Thallium(I) on Growth, Nutrient Absorption, Photosynthetic Pigments, and Antioxidant Response of *Dittrichia* Plants

**DOI:** 10.3390/antiox12030678

**Published:** 2023-03-09

**Authors:** Francisco Espinosa, Alfonso Ortega, Francisco L. Espinosa-Vellarino, Inmaculada Garrido

**Affiliations:** Research Group FBCMP(BBB015), Faculty of Sciences, Campus Avenida de Elvas s/n, University of Extremadura, 06006 Badajoz, Spain

**Keywords:** antioxidant defense system, ascorbate, *Dittrichia*, glutathione, hydrogen sulfide, nitric oxide, photosynthesis, reactive oxygen species, thallium toxicity

## Abstract

*Dittrichia* plants were exposed to thallium (Tl) stress (10, 50, and 100 µM) for 7 days. The Tl toxicity altered the absorption and accumulation of other nutrients. In both the roots and the leaves, there was a decline in K, Mg, and Fe content, but an increase in Ca, Mn, and Zn. Chlorophylls decreased, as did the photosynthetic efficiency, while carotenoids increased. Oxidative stress in the roots was reflected in increased lipid peroxidation. There was more production of superoxide (O_2_^.−^), hydrogen peroxide (H_2_O_2_), and nitric oxide (NO) in the roots than in the leaves, with increases in both organs in response to Tl toxicity, except for O_2_^.−^ production in the roots, which fluctuated. There was increased hydrogen sulfide (H_2_S) production, especially in the leaves. Superoxide dismutase (SOD), ascorbate peroxidase (APX), dehydroascorbate reductase (DHAR), monodehydroascorbate reductase (MDHAR), and glutathione reductase (GR) showed increased activities, except for APX and MDHAR in the roots and GR in the leaves. The components of the ascorbate–glutathione cycle were affected. Thus, ascorbate (AsA) increased, while dehydroascorbate (DHA), reduced glutathione (GSH), and oxidized glutathione (GSSG) decreased, except for in the roots at 100 µM Tl, which showed increased GSH. These Tl toxicity-induced alterations modify the AsA/DHA and GSH/GSSG redox status. The NO and H_2_S interaction may act by activating the antioxidant system. The effects of Tl could be related to its strong affinity for binding with -SH groups, thus altering the functionality of proteins and the cellular redox state.

## 1. Introduction

Plants’ response to stress includes a sharp increase in reactive oxygen, nitrogen, and sulfur species (ROS, RNS, and RSS, respectively) production, which alters the cellular redox balance [1,2,3,4]. The damage that is induced by this alteration, and by the excess ROS, is dealt with by the enzymatic and non-enzymatic antioxidant systems [5,6,7]. The former includes superoxide dismutase (SOD, EC 1.15.1.1), peroxidases (POD, EC 1.11.1.7), catalases (CAT, EC 1.11.1.6), ascorbate peroxidase (APX, EC 1.11.1.11), glutathione reductase (GR, EC 1.6.4.2), monodehydroascorbate reductase (MDHAR, EC 1.6.5.4), and dehydroascorbate reductase (DHAR, EC 1.6.4.2), and the latter includes ascorbate (AsA), reduced glutathione (GSH), phenolics, alkaloids, non-protein amino acids, α-tocopherols, and carotenoids [2,8]. The ascorbate–glutathione (AsA–GSH) cycle removes hydrogen peroxide (H_2_O_2_), maintaining redox homeostasis [2]. The production of nitric oxide (NO), the main RNS, is also related to plants’ response to stress [9,10,11]. It acts at the level of the expression of the defense genes that are involved in eliminating ROS [12,13,14] and plays a key role in the defense mechanisms against different stressors [15], including heavy metals, such as Cd and As [9,16,17,18,19]. Heavy metals increase the synthesis of NO [9,16,20], which is involved in activating the antioxidant defense system and in eliminating the excess ROS that is produced, thus contributing to the maintenance of redox homeostasis [9,21,22,23]. Hydrogen sulfide (H_2_S) is involved in numerous metabolic processes in plants [24,25,26], including the response to heavy metal toxicity [20,25,27,28]. The exogenous application of H_2_S enhances the antioxidant defense system’s response, allowing the oxidative stress that is induced by heavy metals to be reduced [29,30]. H_2_S modulates the activation of the antioxidant system and the gene expression of its components [31,32]. Both NO and H_2_S act by controlling the AsA–GSH cycle components and the ROS levels, efficaciously removing H_2_O_2_ [33]. The levels of GSH are key in the processes of defense against heavy metals, being able to bind to them and also contribute to the biosynthesis of phytochelatins [27,34]. Through the induction of the antioxidant system, the interaction between H_2_S and GSH lowers ROS production under conditions of heavy metal toxicity [29,35,36]. The increase in NO and H_2_S, and their interaction, may act on the antioxidant systems that are involved in the stress response, enhancing their activity and the expression of the genes that are involved in these antioxidant defense systems, thus contributing to the elimination of ROS and the maintenance of redox homeostasis [26,32,37].

Thallium (Tl) is a very toxic element for living beings. It belongs to group IIIA of the periodic table, occurring as Tl(I) and Tl(III). The former is the most stable form, and the latter is the most toxic [38]. Tl forms minerals, including silicates and sulfates [39]. It is widely distributed in the environment in low concentrations of between 0.3 and 0.5 µg g^−1^ [40]. It is used as a catalyst in alloys, optical lenses, jewelry, low-temperature thermometers, semiconductors, dyes, etc., and its salts as rodenticides and insecticides; although, the WHO recommends against its use due to its great toxicity [40]. It is found in ionic form in drainage waters [41,42,43], being released from the aqueous waste and soils of disused Pb–Zn and As mines.

Since Tl and K have similar ionic radii, they can be absorbed by plants through the same mechanisms, depending on the plant species [38]. Tl-induced toxicity is commonly a symptom of the replacement of K with Tl [44]. While in *Arabidopsis thaliana* Tl and K have antagonistic effects in that they mutually interfere with each other’s absorption [45], in *Biscutella laevigata,* increased K does not inhibit the uptake of Tl and, therefore, it seems that the absorption of Tl would not be carried out through the same systems as K, but it will be carried out through specific transporters [46].

Tl hyperaccumulator plants have been identified in the Brassicaceae family [44]. They accumulate Tl in their leaves and roots, and, to a lesser extent, in their stems and fruits, with a dependence on species and soil type. Thus, in *Brassica juncacea,* the maximum Tl accumulation in the aerial part is in the leaves and the minimum in the flowers [39], whereas in other species, Tl accumulation occurs mainly in the roots, then the leaves, and, to a lesser extent, in the stem [47,48,49]. Some ferns and aquatic plants of the genus *Lemna* are reported to have a great capacity for Tl accumulation, which would make them interesting for use in Tl phytoremediation processes [42,43].

Various hydroponic culture studies have been carried out on the absorption, accumulation, and toxicity of Tl [46,50,51]. In *Sinapis alba* under these conditions, most of the Tl is transported to the leaves [50]. In *Silene latifolia* and *Biscutella laevigata*, low Tl concentrations induce a hormetic increase in biomass in response to slight toxic stress [46,51,52,53]. This contrasts with the results of the in vitro culture of *Arabidopsis,* which show a decline in biomass [45]. Although Tl(I) is not a redox metal, it can alter photosynthetic electron transport, leading to increased ROS and MDA [49]. The oxidation of pigments, the alteration of complexes, and the disappearance of the grana occur in the discolored foliar areas [44]. Chang et al. [45] describe the inhibition of gene expression of LCH II subunits. Tl toxicity induces oxidative stress [44], with increases in H_2_O_2_ and in SOD, APX, and POX activities [49,54]. These enzymes are also positively regulated at the gene expression level under Tl toxicity [44]. The effect differs between the ionic forms, with Tl(III) inducing greater ROS production than Tl(I) [55]. Pu et al. [48] describe a decrease in the SOD activity and an increase in POX, while Liu et al. [56] describe increases in both of these activities. This activity in response to heavy metal toxicity can vary with exposure time and can also inhibit the synthesis of these enzymes and alter their assembly [57]. Tl has a strong affinity for the amino imino and the sulfhydryl groups of proteins and other biological macromolecules, an example being glutathione, which decreases in its reduced form (GSH) due to oxidation or to the formation of complexes with Tl (Tl(SG)_3_) [27,34]. There has yet to be evidence for the participation of NO and H_2_S in these defense responses. *Dittrichia viscosa* (L.) Greuter is a species belonging to the Asteraceae family that develops in a wide range of soils and climatic conditions in the Mediterranean basin. *Dittrichia* can colonize poor soils and also those that are degraded due to anthropic activities, such as mining, with high concentrations of heavy metals and metalloids [58]. *Dittrichia* are capable of absorbing and accumulating large amounts of Cd, Cu, Fe, Ni, Pb, Zn, As, and Sb [11,58,59,60,61,62,63,64]. The participation of the components of the AsA–GSH cycle, as well as NO and H_2_S, in the Sb accumulation process has recently been reported [11,63]. However, this capacity varies depending on the different genotypes or populations, which shows great genetic plasticity [60,61,62]. In accordance with this, *Dittrichia* can be considered a good candidate to carry out phytoremediation processes for heavy metals and metalloids, despite not being a hyperaccumulator plant [62]. The present study was therefore aimed at determining the involvement of ROS, NO, H_2_S, and the antioxidant systems in *Dittrichia* plants’ response to Tl, and the morphophysiological alterations that areinduced by the toxicity of this element.

## 2. Materials and Methods

### 2.1. Plant Materials, Growth Conditions, and Treatments

The seeds of *Dittrichia viscosa* (L.) Greuter obtained from “Semillas Silvestres, SL” were surface sterilized for 15 min in 10% sodium hypochlorite solution (40 g L^−1^), rinsed several times with distilled water, and, before their germination, were imbibed in distilled water, aerated, and agitated for 2 h at room temperature. After imbibition, the seeds were germinated in a plastic container (30 × 20 × 10 cm) filled with a sterilized perlite mixture substrate wetted with Hoagland solution and were kept at 27 °C in the dark for 48 h. After germination, the seedlings were cultivated for 5 days at 27 °C and 85% relative humidity, with a light/dark photoperiod of 16 h/8 h, and a constant illumination under photosynthetic photon flux density of 350 μmol m^−2^ s^−1^ during the day.

After 7 days, the plants were grown in hydroponic culture in lightweight polypropylene trays (20 × 15 × 10 cm; 4 plants per container) and the same environmental conditions (except for relative humidity, 50%). The plants were cultivated in a basal nutrient solution composed of the following: 4 mM KNO_3_, 3 mM Ca(NO_3_)_2_ 4H_2_O, 2 mM MgSO_4_ 7H_2_O, 6 mM KH_2_PO_4_, 1 mM NaH_2_PO_4_ 2H_2_O, 10 μM ZnSO_4_ 7H_2_O, 2 μM MnCl_2_ 4H_2_O, 0.25 μM CuSO_4_ 5H_2_O, 0.1 μM Na_2_MoO_4_ 2H_2_O, 10 μM H_3_BO_3_, and 20 μM NaFeIII-EDTA. After 10 days in hydroponic culture, we started our assay with Tl. For the Tl treatment, the basal solution was supplemented with Tl (I) sulfate (Tl_2_SO_4_) in final concentrations of 0 µM (control), 10 µM, 50 µM, and 100 µM Tl. Each cultivation solution was adjusted to pH 5.8, continuously aerated, and changed every 5 days. The plants were exposed to the Tl treatments for 7 days.

The plants of each treatment were divided into roots and shoots, which were washed with distilled water, dried on filter paper, and weighed to obtain the fresh weight (FW). Half of the roots and shoots from each Tl treatment were dried in a forced-air oven at 70 °C for 24 h to obtain the dry weight (DW) and the subsequent analysis of the concentration of Tl. The other half of the fresh roots and leaves were used for the biochemical analyses. The relative water content (RWC) of the leaves was determined at the time of harvest from fresh material in accordance with the method described by Smart and Bingham [65]. Leaf disks were collected from the different treatments, and their FWs were determined. They were then immersed in distilled water for 1 h, dried externally with filter paper, and weighed again to obtain the turgid weight (TW). Finally, they were oven-dried at 70 °C for 24 h and weighed to obtain the DW. The RWC was calculated as RWC% = (FW − DW)/(TW − DW) × 100.

### 2.2. Determination of Tl and Mineral Content

The plant material (roots and leaves) of the control and Tl treatments was harvested and rinsed with distilled water. After 24 h of drying at 70 °C, the root and leaf material was crushed in a marble ceramic mill. The Tl, K, Mg, Ca, Fe, Mn, Cu, and Zn content was measured by inductively coupled plasma-mass spectrometry (ICP-MS, model NexION 300, PerkinElmer) in accordance with Lehotai et al. [66]. The bioaccumulation factor (BF) was calculated from the ratio between the concentration of the element in the roots or leaves and that present in the hydroponic solution, and the de translocation factor (TF) was calculated from the ratio between the concentration of the element in the leaves and in the roots.

### 2.3. Determination of the Soluble Amino Acid and Protein Contents

The fresh plant material (roots and leaves) of the control and Tl treatments was homogenized (0.2 g mL^−1^) in 50 mM sodium phosphate buffer, pH 7.0, then filtered through muslin, centrifuged at 12,360× *g* for 15 min, and the supernatant was used for the protein and amino acid assays. For the protein assay, Bradford’s method was used [67]. The results are expressed as mg g^−1^ FW against an albumin standard curve. For the amino acid assay, the method of Yemm and Cocking [68] was used, with ninhydrin reagent. The results are expressed as mg g^−1^ FW against a glycine standard curve.

### 2.4. Determination of Photosynthetic Pigment Contents and Photosynthetic Efficiency

The chlorophyll and carotenoid contents of the leaves were determined at the end of each trial. About 0.125 g of fresh leaves were incubated in 10 mL methanol for 24 h in the dark. The concentrations of chlorophylls and carotenoids were measured spectrophotometrically (Shimadzu UV1603) at A_666_, A_653_, and A_470_. The total chlorophyll and carotenoid content was calculated as described by Wellburn [69] and expressed as µg g^−1^ FW.

For the determination of the photosynthetic parameters, the middle region of the fully expanded upper leaves at the end of each Tl treatment were adapted in the dark for 10 min, and then the minimal fluorescence (F_0_), the maximal chlorophyll fluorescence (F_m_), and the maximum photosynthetic efficiency (F_v_/F_m_) were recorded with a handheld fluorometer (Chlorophyll Fluorometer, OS-30p, Opti-Sciences). The variable fluorescence (F_v_) and the rate constants of photochemical and nonphotochemical deactivation of excited Chl molecules (F_v_/F_0_) was calculated.

### 2.5. Determination of Lipid Peroxidation and Reactive Oxygen Species (O_2_^.−^ and H_2_O_2_), NO, and H_2_S Contents, and SOD Activity

To analyze oxidative stress, the formation of malondialdehyde (MDA) was determined using thiobarbituric acid (TBA). Briefly, 0.25 g of plant material (roots or leaves) was homogenized with 2.5 mL of solution containing 0.25% TBA and 10% trichloroacetic acid (TCA). The mixture was incubated at 95 °C for 30 min. The reaction was stopped by immersing the tubes in ice, then filtering, and centrifuging at 8800× *g* for 10 min. The MDA was determined spectrophotometrically in the supernatant at A_532_−A_600_ with ε = 155 mM^−1^ cm^−1^ and expressed as μmol MDA g^−1^ FW [70]. The H_2_O_2_ content was analyzed in accordance with Velikova et al. [71]. Fresh material (roots or leaves) was homogenized (0.2 g mL^−1^) in 0.1% TCA. The homogenate was centrifuged at 12,000× *g* for 15 min. The reaction mixture contained 0.5 mL of supernatant, 0.5 mL 10 mM of potassium phosphate buffer (pH 7.0), and 1 mL of 1 M KI solution. The H_2_O_2_ concentration was estimated based on the reaction mixture absorbance at A_390_ using a standard curve of H_2_O_2_. The O_2_^.−^ generating and SOD activities were measured in an extract obtained from roots or leaves that was homogenized (0.5 g mL^−1^) at 4 °C in 50 mM phosphate buffer, pH 6.0, 0.5 mM phenylmethylsulfonyl fluoride (PMSF), 1 mM β-mercaptoethanol, and 1 g L^−1^ plyvinylpolypyrrolidone (PVPP). The homogenate was filtered and centrifuged at 39,000× *g* for 30 min at 4 °C, and the supernatant was collected as an enzyme extract. The O_2_^.−^ generation was assayed spectrophotometrically by measuring the oxidation of epinephrine to adrenochrome at A_480_ (ε = 4.020 mM^−1^ cm^−1^) [72]. The reaction mixture contained 1 mM epinephrine in acetate buffer 25 mM, pH 5.0. The SOD activity was determined in 50 mM phosphate buffer, pH 7.8, 0.1 mM ethylene diamine tetra-acetic acid (EDTA), 1.3 μM riboflavin, 13 mM methionine, and 63 μM nitro blue tetrazolium (NBT) [73]. The reaction mixture was maintained in the dark at 25 °C, and the reaction was started by the addition of the riboflavin and enzyme extract and was illuminated for 2 min. The changes in absorbance A_560_ were measured. A unit of SOD is defined as the amount of enzyme required to cause 50% inhibition of NBT reduction. The NO content was determined using the method described by Zhou et al. [74]. Roots or leaves (0.5 g) were homogenized in 3 mL of 50 mM cool acetic acid buffer (pH 3.6, containing 4% zinc diacetate). The homogenates were centrifuged at 10,000× *g* for 15 min at 4 °C. The supernatant was collected. The pellet was washed in 1 mL of acetic acid buffer and centrifuged as before. The two supernatants were combined, and charcoal was added, followed by vortexing and filtration. A mixture of filtrate and the Griess reagent (1:1) was incubated at room temperature for 30 min. The absorbance was determined at A_540_. The NO content was calculated by comparison against a standard curve of NaNO_2_ and expressed as nmol NaNO_2_ g^−1^ FW. The H_2_S concentration was determined using Li’s method [75]. Roots or leaves were ground in liquid nitrogen, homogenized (0.5 g mL^−1^) in 20 mM Tris-HCl buffer, pH 8.0, containing 10 mM EDTA and 20 mM Zn(OAc)_2_, and centrifuged at 15,000× *g* for 15 min at 4 °C. The supernatant was combined with 30 mM FeCl_3_ (in 1.2 M HCl) and 20 mM DMPD (in 7.2 M HCl) (1:1:1). The mixture was incubated at room temperature for 15 min and the A_670_ was determined. The H_2_S content was calculated by comparison against a standard curve of NaHS and expressed as nmol H_2_S g^−1^ FW.

### 2.6. Determination of the Components and Antioxidant Enzymes of the AsA–GSH Cycle

To determine the total ascorbate and glutathione, fresh roots or leaves (1 g mL^−1^) were homogenized at 4 °C in 5% metaphosphoric acid. The homogenate was centrifuged at 20,000×× *g* for 20 min at 4 °C, and the supernatant was collected for the determination of ascorbate and glutathione. The total ascorbate pool (AsA + DHA) and total glutathione pool (GSH + GSSG) were determined in accordance with De Pinto et al. [76]. Total ascorbate was determined by the reduction of DHA to AsA, and the concentration of DHA was estimated from the difference between the total ascorbate pool and the AsA. The ascorbate pool was determined at A_525_. The glutathione pool was determined from the change in absorbance at A_412_ over 1 min. GSH was estimated as the difference between the amount of total glutathione pool and that of GSSG.

To determine the activities of the enzymes involved in the AsA–GSH cycle—APX, MDHAR, DHAR, and GR—the roots or leaves (0.5 g L^−1^) were homogenized at 4 °C in 50 mM phosphate buffer, pH 7.5, 0.5 mM PMSF, 1 mM β-mercaptoethanol, 1 g L^−1^ PVPP, and 5 mM AsA for the APX activity. The homogenate was filtered and centrifuged at 39,000× *g* for 30 min at 4 °C, and the supernatant was used for the enzyme determinations. The APX activity was determined spectrophotometrically by measuring the oxidation of ascorbate at A_290_ for 2 min (ε = 2.8 mM^−1^ cm^−1^) [77]. The reaction mixture contained 0.5 mM ascorbate, 0.2 mM H_2_O_2_, and the enzyme extract, at 25 °C, in 0.1 M phosphate buffer, pH 7.5, and EDTA 0.5 mM, expressing the result as μmol ascorbate min^−1^ mg^−1^ protein. The DHAR activity was determined from the oxidation of DHA at A_265_ for 1 min (ε = 14 mM^−1^ cm^−1^) [78] in a medium containing 0.1 M phosphate buffer (pH 6.5), 0.5 mM EDTA, 2.5 mM GSH, 0.5 mM DHA, and the enzyme extract. The DHAR activity is expressed as nmol ascorbate min^−1^ mg^−1^ protein. The MDHAR activity was determined from the oxidation of NADH at A_340_ for 1 min (ε = 6.22 mM^−1^ cm^−1^) [79] in a medium containing 50 mM Tris-HCl buffer (pH 7.8), 10 mM AsA, 0.2 mM NADPH, 0.5 units of ascorbate oxidase, and the enzyme extract, expressing the result as μmol NADH min^−1^ mg^−1^ protein. The GR activity was determined at A_340_ from the oxidation of NADPH for 3 min (ε = 6.22 mM^−1^ cm^−1^) [78] in a medium containing 0.1 M phosphate buffer (pH 7.5), 0.5 mM EDTA, 0.5 mM GSSG, 0.2 mM NADPH, and the enzyme extract, expressing the result as nmol NADPH min^−1^ mg^−1^ protein.

### 2.7. Statistical Analyses

The data to be presented are the means ± SE of at least 10 replicates obtained from three independent experiments. For each measurement, a Shapiro–Wilk normality test was performed (since *n* < 50) to verify if they had a normal distribution. Later, we applied a parametric test of one-way ANOVA. Those values where there are significant differences are marked with different letters, that is, where *p* ≤ 0.05. All statistical analyses were performed with Microsoft Excel 365 (Microsoft Inc., Alburquerque, NM, USA) and the SPSS v. 24 package (SPSS Inc., Chicago, IL, USA).

## 3. Results

### 3.1. Effect of Tl on the Growth, Accumulation of Mineral Elements, and Soluble Amino Acid and Protein Content of Dittrichia

**Figure 1:**
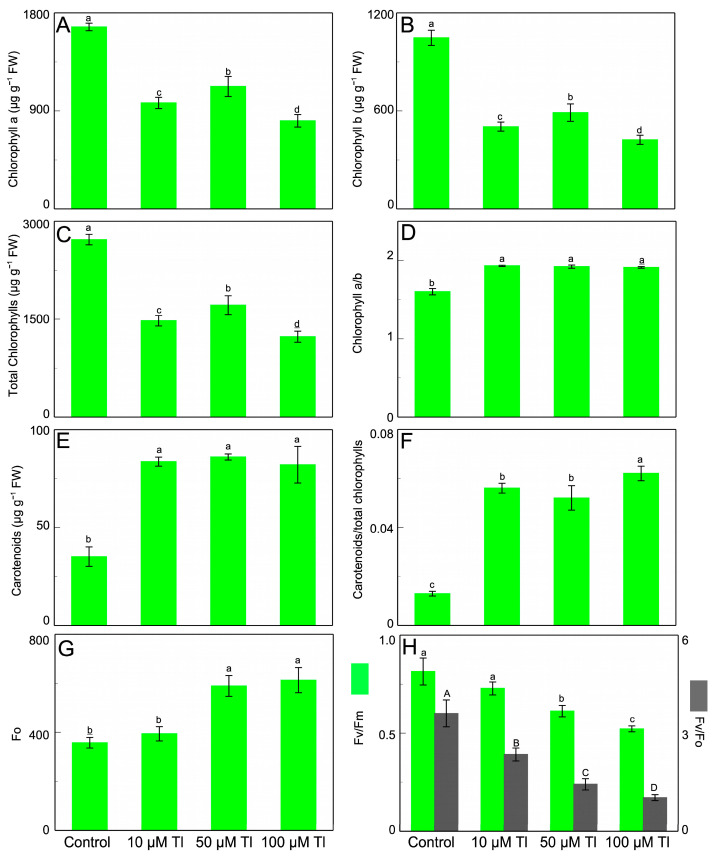
Effect of Tl on photosynthetic pigments and parameters in leaves of *D. viscosa*. Chlorophyll a content (**A**), chlorophyll b content (**B**), total chlorophylls (**C**), chlorophyll a/b (**D**), carotenoids content (**E**), carotenoids/total chlorophylls (**F**), F_o_ (**G**), and F_v_/F_m_ and F_v_/F_o_ (**H**). The data are the means ± SE from ten independent experiments. The different letters indicate significant differences at *p* < 0.05.

The growth of *Dittrichia* is very similar at all of the Tl concentrations, with little difference between them (Figure 1A, Table 1). On the contrary, the growth that can be observed in the control plants, with 0 µM Tl, is much greater. The plants that were grown under Tl toxicity show a significantly smaller size than the control plants in both the shoot and the root parts (Table 1); furthermore, the leaves appear chlorotic, especially in the venal area and, to a lesser extent, in the interveinal area. The length of the shoots decreases drastically with the Tl concentration to values of 38% and 32% for 10 µM and 100 µM, respectively. The effect of the three concentrations of Tl on the growth of the roots is similar, with an inhibition of 48% with respect to the length of the control roots.

The fresh weight of both the shoots and the roots decreases in response to the Tl dose. This weight decreases significantly in the shoots as the Tl concentration increases (73%, 77%, and 82% for 10 µM, 50 µM, and 100 µM Tl, respectively). On the contrary, in the roots, the fresh weight is similar in the different concentrations of Tl that were used, being 75% of the control values. The dry weight decreases in both the shoots and the roots, depending on the concentration of Tl in the medium. The DW/FW percentage increases in the plants that were subjected to Tl toxicity, with the greatest value in the shoots being observed at 100 µM Tl. In the roots, an increase in this percentage can be observed due to the effect of Tl, although this increase is less when increasing the Tl. The RWC (Table S1) shows no alteration in the roots, while it decreases very slightly in the shoots (≈9%).

The absorption and the accumulation of Tl increases with the concentration of Tl in the medium (Table 2). In the roots, the Tl content is positively correlated with that of the medium, but not in leaves, where the accumulation of Tl is similar for 50 µM and 100 µM. The accumulation observed here is much greater in the roots than in the shoots. Thus, in the treatment with 10 µM Tl, the accumulation in the roots is 6× that produced in the leaves, with this accumulation being of greater magnitude with the higher Tl concentrations. This greater capacity for accumulation in the roots is reflected in a higher BF (i.e., BF_roots_ > 350, BF_shoots_ < 100). On the other hand, the value of TF_Tl_ is low, decreasing progressively with the increase in Tl in the medium. As the concentration of Tl in the roots increases, its transport capacity decreases (from 0.163 to 0.043 for 10 µM and 100 µM Tl, respectively).

Table 3 shows the effect of Tl toxicity on the absorption and the accumulation of different macroelements (K, Mg, and Ca) and microelements (Fe, Mn, Cu, and Zn). The K content in the root and shoot samples shows a decreasing trend as the Tl concentration increases, and this decrease is greater in the roots than in the leaves. However, there is a wide variability in the measured data for this microelement. With regard to the value of TF_K_, it is similar to the control value, except with 10 µM Tl, in which a slight decrease can be observed (Table S2). Similarly to K, the Mg content (Table 3) decreases due to Tl independently of the concentration, and this is more pronounced in the roots than in the shoots. The TF_Mg_ values increase (×2) with respect to the control, although they are similar for the three Tl concentrations. The lowest Ca content in the roots is observed in the plants that were grown in 10 µM of Tl, with a decrease of 52%, relative to the control. However, in the roots of the plants that were grown with 50 µM Tl, the decrease is 20%, and with 100 µM Tl, the amount of this element increases (by 28%, relative to the control). In the shoots, the Ca content is not significantly modified.

With regard to the absorption and the accumulation of Fe, Mn, Cu, and Zn (Table 3), in the roots there is an increase in the content of Mn, Cu, and Zn, while the content of Fe decreases. While Cu and Zn increase with an increasing Tl dose, the increase in Mn is similar for all of them. The decrease in the Fe content is smaller in response to an increase in the concentration of Tl in the medium (54%, 45%, and 24% for 10 µM, 50 µM, and 100 µM Tl, respectively). In the leaves, Fe decreases with the increase in Tl in the medium and Cu decreases by 30% (independently of the Tl concentration), while Mn and Zn increase. With regard to the values of TF (Table S2), the TF_Fe_ increases at 10 µM Tl, but is similar to the control value for the other concentrations. The TF_Mn_ increases, while TF_Cu_ and TF_Zn_ decrease.

Tl induces an increase in the amino acid content, which is greater in the leaves (≈×4.3) than in the roots (≈×1.8) (Table 4). This strong increase does not depend on the dose of Tl that is used. With regard to the protein content (Table 4), in the roots that were subjected to 10 µM of Tl toxicity, there is little difference from the control, with a slight increase being observed; however, with 50 µM and 100 µM, a progressive decrease can be observed, being by 16% and 41%, respectively. In the leaves, there is a decrease, which is greatest with 10 µM Tl (by 43%, relative to the control).

### 3.2. Effect of Tl on Photosynthetic Pigment Content and Photosynthetic Efficiency

**Figure 2:**
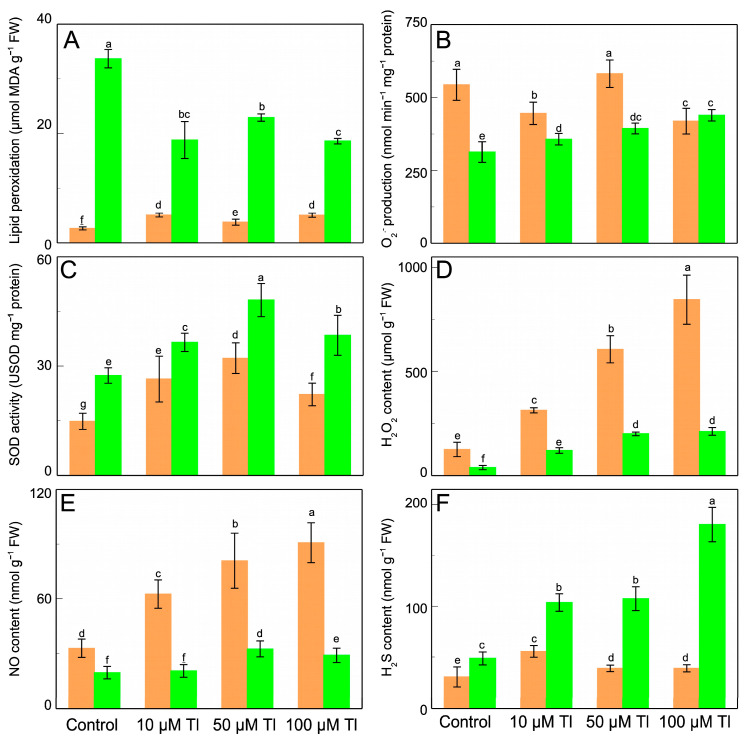
Effect of Tl on the lipid peroxidation (**A**), O_2_.^–^ production (**B**), SOD activity (**C**), and H_2_O_2_ (**D**), NO (**E**), and H_2_S (**F**) content in roots (orange) and leaves (green) of *D. viscosa*. The data are the means ± SE from ten independent experiments. The different letters indicate significant differences at *p* < 0.05.

Tl toxicity induces a decrease in both chlorophyll a and b, and, consequently, in total chlorophyll (Figure 1A–C). The decrease in these contents is smaller and very similar in 10 µM and 50 µM, with values of 54% and 63% of the control, respectively. For 100 µM Tl, this content is 45% of the control. The chlorophyll a/b ratio (Figure 1D) increases in response to Tl, going from 1.60 to 1.92 for the control leaves and the leaves that were subjected to Tl, respectively. In contrast, the carotenoid content (Figure 1E) shows a clear increase (by around 140%) in the plants that were subjected to Tl, although with no difference between the different concentrations that were used. The decrease in chlorophylls and the increase in carotenoids caused by Tl translate into an increase in the carotenoids/chlorophylls ratio (Figure 2F).

In the Tl treatments, the F_0_ value increases as the Tl concentration increases (Figure 1G). Thus, the F_0_ increases by 10% for 10 µM Tl, while with 50 µM and 100 µM Tl the increases are much greater, at 65% and 71%, respectively. The efficiency of photosystem II (F_v_/F_m_) (Figure 1H) is also strongly affected by the Tl doses, with the decreases being dependent on the increase in Tl, ranging from 10% to 36% for 10 µm and 100 µM Tl, respectively. With regard to the F_v_/F_0_ ratio (Figure 1H), there is a marked decrease with the increase in the Tl concentration that was used. Thus, while in the control plants the F_v_/F_0_ ratio is 3.604, in those that were treated with 100 µM this ratio is 1.026.

### 3.3. Lipid Peroxidation, Production of O_2_^.−^ and SOD Activity, and H_2_O_2_, NO, and H_2_S Content

Figure 2 shows the response to the treatments with Tl at the level of lipid peroxidation production of O_2_^.−^ and SOD activity, and H_2_O_2_, NO, and H_2_S content. In the roots, lipid peroxidation increases in response to Tl (Figure 2A). On the contrary, in the leaves, the levels of lipid peroxidation are lower, with similar values for the three concentrations of Tl that were used. The production of O_2_^.−^ (Figure 2B) also differs between the roots and the leaves, being greater in the former. In the roots, the differences between the treatments are slight and varying, with decreases of 18% and 23% for 10 µM and 100 µM Tl, respectively, but with no significant change for 50 µM Tl. In the leaves however, the O_2_^.−^ production increases with increasing Tl concentrations in the medium. The SOD activity, in response to Tl (Figure 2C), increases in both the roots and the leaves, with the latter presenting the greatest values. In both of the organs, the SOD activity increases for all of the concentrations of Tl. For the treatment with 10 µM Tl, the SOD activity increases by ×1.8 and ×1.4 for the roots and the leaves, respectively, and for 50 µM Tl, by ×2.3 and ×1.8, respectively. With 100 µM of Tl, the SOD activity also increases, although at lower values (×1.6 and ×1.4, in the roots and the leaves, respectively). In both of these organs, the lowest and highest Tl concentrations cause similar increases in SOD activity. The H_2_O_2_ content (Figure 2D) increases in both the roots and the leaves in response to Tl-induced stress. The H_2_O_2_ content in the roots increases from 125 µmol g ^–1^ FW in the controls to values that reach 844 µmol g^−1^ FW for 100 µM Tl and shows a strong positive correlation with the Tl concentration. The relative increases are ×2.5, ×4.8, and ×6.7 for 10 µM, 50 µM, and 100 µM Tl, respectively. A similar response is seen in the leaves, although in this case the levels of H_2_O_2_ stabilize at 50 µM Tl, with values for 50 µM and 100 µM being similar. The production of NO (Figure 2E) is much greater in the roots than in the leaves, and it increases in response to the increase in Tl treatments. Thus, 10 µM of Tl induces a ×1.8 increase, with the increases for the two greater concentrations being very similar to each other (×2.5 and ×2.6, respectively). In the leaves, there are also increases in response to Tl toxicity, by very similar factors (≈×1.25) for all of the concentrations. With regard to the production of H_2_S (Figure 2F), the levels are higher in the leaves than in the roots. In the roots, the greatest increase in H_2_S content corresponds to the treatment with 10 µM Tl (×1.8). The greater Tl concentrations induce only slight changes in the H_2_S content, with values that are very similar to the control. In the leaves, there is increased H_2_S content for all of the concentrations, with the maximum value corresponding to the 100 µM Tl treatment (×3.7, relative to the control).

### 3.4. APX, DHAR, MDHAR, and GR, and Ascorbate and Glutathione Pool

**Figure 3:**
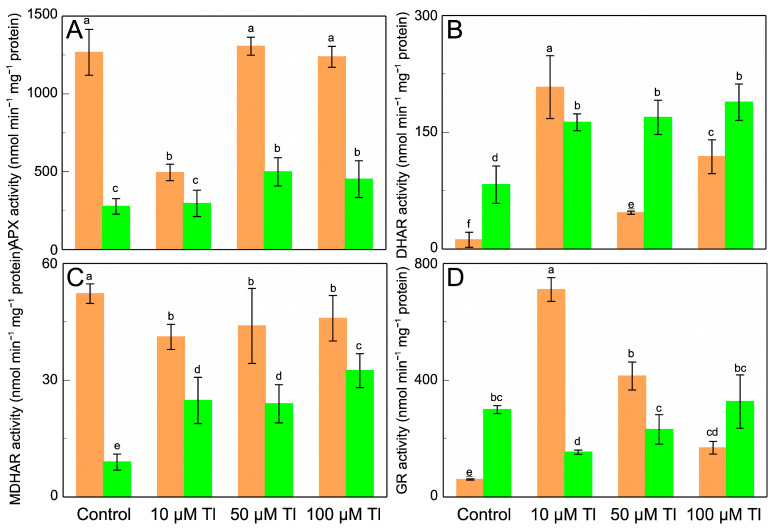
Effect of Tl on the APX (**A**), DHAR (**B**), MDHAR (**C**), and GR (**D**) activities in roots (orange) and leaves (green) of *D. viscosa*. The data are the means ± SE from ten independent experiments. The different letters indicate significant differences at *p* < 0.05.

The effect that Tl has on the enzymes of the AsA–GSH cycle was determined (Figure 3). APX, DHAR, MDHAR, and GR all present behavior that differs between the roots and the leaves. In the roots, the APX activity (Figure 3A) decreases after the stress that is induced by 10 µM Tl but is unaltered by 50 µM or 100 µM Tl. On the contrary, in the leaves, the APX activity increases at the higher Tl concentrations, but is unaffected by 10 µM Tl. The DHAR activity in the roots increases strongly with 10 µM Tl, but the greater concentrations show less activation (Figure 3B). In the leaves, DHAR increases by very similar factors (≈×2.0, relative to the control) in response to all of the Tl concentrations. The MDHAR activity (Figure 3C) also differs in behavior between the roots and the leaves; while in the roots there are slight decreases in this activity at 10 µM, in the leaves there are increases (by between ×2.8 and ×3.7) for all of the Tl concentrations, as was the case for the DHAR activity. The GR activity (Figure 3D) increases in the roots, but this increase is less the greater the Tl concentration (1.8- and 5.4-fold for 10 µM and 100 µM Tl, respectively). In the leaves, the GR activity decreases with 10 µM and 50 µM Tl but is not significantly altered with 100 µM Tl.

**Figure 4:**
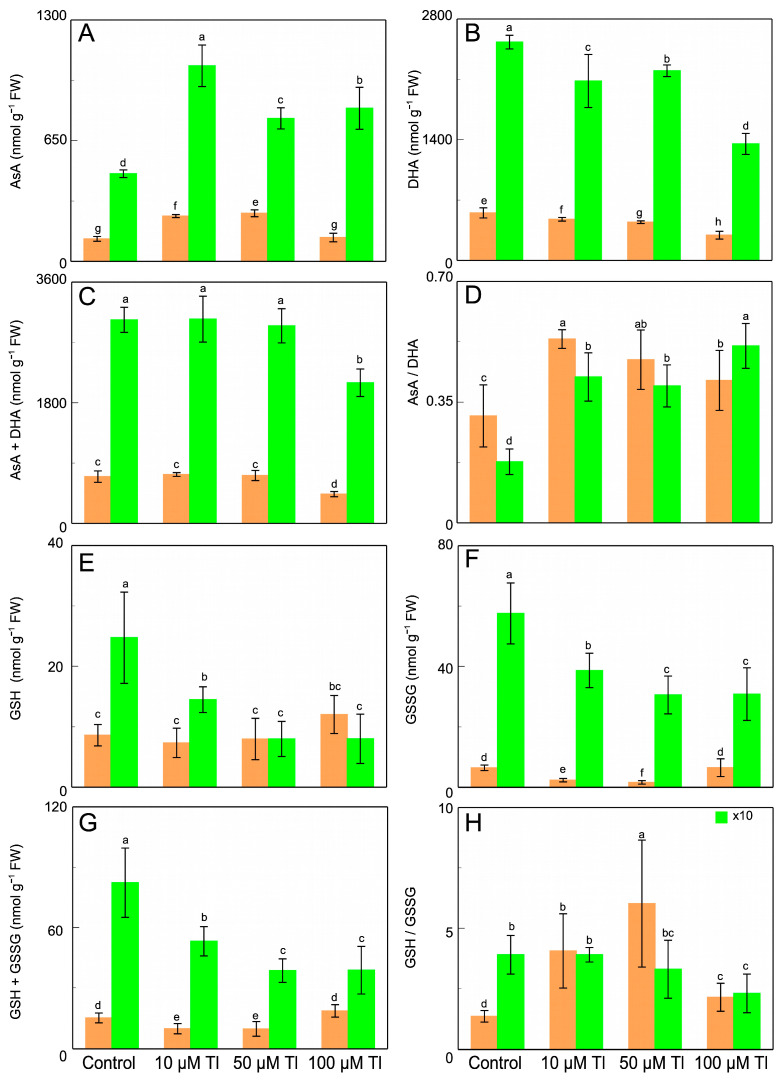
Effect of Tl on the AsA (**A**), DHA (**B**), and ascorbate pool (AsA + DHA) (**C**) contents; the AsA/DHA ratio (**D**), the GSH (**E**), GSSG (**F**), and glutathione pool (GSH + GSSG) (**G**) contents; and the GSH/GSSG ratio (H), in *D. viscosa*. The data are the means ± SE from ten independent experiments. The different letters indicate significant differences at *p* < 0.05.

The AsA and DHA contents are altered by Tl (Figure 4A,B). Thus, in the roots, the control AsA content is 121 nmol g ^−1^ FW, and Tl duplicates (×2.0) this content for 10 µM and 50 µM Tl, while 100 µM does not alter from the control value. On the contrary, the DHA content decreases by 14–48%, relative to the control, depending on the Tl concentration. In the leaves, the result is very similar, although the contents of both AsA and DHA are greater than those in the roots. The increase in AsA content is 2.23-fold in the presence of 10 µM Tl, and 1.63- and 1.49-fold for 50 µM and 100 µM Tl, respectively. The DHA content decreases for all of the concentrations. The total ascorbate pool (AsA + DHA) (Figure 4C) is unaltered, except with 100 µM Tl, which reduces this pool in both of the organs. The redox state (AsA/DHA) (Figure 4D) is altered, increasing in the roots and the leaves, in response to the Tl concentrations. With regard to the GSH content (Figure 4E), in the roots there is an increase only in those that were subjected to 100 µM Tl, but not for the other concentrations. The GSSG content (Figure 4F) decreases for the two lower concentrations, but not for 100 µM Tl, which produces levels that are similar those of to the control. In the leaves, both the GSH and the GSSG content (Figure 4G) decrease with the Tl concentration, with very similar values for 50 µM and 100 µM Tl. With regard to the glutathione pool, in the roots that were subjected to 10 µM and 50 µM Tl, there is a reduction of 35% of the total content, relative to the control. On the contrary, 100 µM causes a 23% increase (1.23-fold) in the AsA + DHA pool. Whereas, in the leaves, this pool decreases for all of the concentrations of Tl that were used. The GSH/GSSG ratio (Figure 4H) increases in the roots from 1.32 in the control to 4.06, 6.02, and 2.14 for 10 µM, 50 µM, and 100 µM, respectively. In the leaves, this ratio presents a different behavior, decreasing with Tl.

## 4. Discussion

In *Dittrichia viscosa*, the concentrations of Tl that were used here cause a decrease in growth and biomass production (Figure S1, Table 1 and Table S1), with these results being similar to those that have been observed in other plants [45,47,48,80,81,82]. The effect of Tl on the dry matter content and RWC is not significant. Only in the shoots is a slight decrease in RWC observed, a result that is very similar to that described by Radić et al. [49]. This contrasts with the decreases in RWC that has been observed in plants stressed by Cd [83,84,85].

The Tl that is absorbed accumulates mostly in the roots [48,49,86,87], with high values of BF_Tl_, and decreases in TF_Tl_ with an increasing dose of Tl in the medium (Table 2). This preferential accumulation in the roots may obviate more serious damage to photosynthetic pigments [49].

Tl toxicity produces alterations in the absorption and accumulation of other elements (Table 3). There is a tendency to decrease the K content, although this is not significant. This variability may be due to the great genetic variability that is evidenced in *Dittrichia* [61,62]. Thus, the decline in K content is similar to that described by Buendia-Valverde et al. [88] for *Capsicum annuum* leaves, with decreases in the content at a low Tl concentration and a recovery of control values at a greater concentration. The Tl affects K uptake by the roots, but not its mobility within the plant, with TF_K_ (Table S2) being unaltered and the lower shoot content being due to the decrease in the root concentration rather than any reduced translocation. This effect of Tl on K uptake is a result of their mutual antagonism, since Tl uses K transport systems [34]. Chang et al. [45] also describe the antagonistic effect of Tl and K in *Arabidopsis,* in which Tl interferes with the K uptake. In *Sinapis alba* that are grown in hydroponic culture, the effect of Tl is to reduce the K content in the roots, as well as in the stems and leaves [50]. With regard to Mg, the Tl inhibits its absorption in *Dittrichia*, decreasing the content in the roots. In the leaves, the decrease in the Mg content is less, since there is a significant increase in TF_Mg_. However, despite the increase in TF_Mg_ and the lower effect that is observed on the Mg content in the leaves, this does not prevent the decrease in the chlorophyll content. In contrast, in *S. alba*, Holubick et al. [50] describe low Tl concentrations leading to a decrease in Mg in shoots but not in roots, whereas, in *C. annuum,* the changes in the Mg content depend on the variety [88]. With regard to the Ca content, the data show that there is a decrease in its accumulation in the roots, except for those with the highest concentration that was used. The content of this element in the leaves is unaffected, however. Once again, the effects of Tl on the absorption and accumulation of Ca are diverse, depending, as in the case of Mg, on the variety [88] and the accumulating organ [50]. The microelements Fe, Mn, Cu, and Zn behave in different ways. While Fe decreases in both the roots and the leaves [50], Mn and Zn increase in both of these organs, and Cu only increases in the roots but decreases in the leaves. The inhibited growth that is induced by Tl stress may be due to this alteration in the absorption of essential mineral nutrients [89]. Our results show decreases in the absorption of K, Mg, and Fe, and alterations in the absorption and the accumulation of other essential elements, such as Ca, Mn, Cu, and Zn, which have increased presence in both the roots and the leaves, except for Cu, which is below the control levels in the leaves (Table 3 and Table S2).

The increase in amino acid content in response to Tl toxicity could be associated with the observed decrease in the protein content, as well as with an accumulation of amino acids in response to this toxicity [90,91,92,93]. The increase in the soluble amino acid content and the decrease in the protein content show a clear effect of Tl toxicity (Table 4). Similar results have been described in different plants that were subjected to toxicity, not only of Tl [81], but also of other heavy metals, such as Cu, Cd, and Ni [94,95,96]. The decrease in protein content could be due to the effect of oxidative damage on protein synthesis, to alterations at the DNA level that would affect protein synthesis, or to the inhibition of the absorption of other elements, such as Mg and K [94]. It could also be due to increases in protease activity [97]. The increase in amino acid accumulation could be due to protein turnover increasing proteolysis, as well as due to the de novo synthesis of osmoprotectant amino acids, which is usually the case for stress-induced proline accumulation [90,91,93]. The amino acids can act on osmoregulation, heavy metal detoxification, and the formation of polyamines and glutathione, secondary metabolites, or the organic nitrogen reserve [92].

The decrease in the chlorophyll content that was observed in *Dittrichia* in response to stress that was induced by Tl toxicity (Figure 1A–D) coincides with results that have been described by other authors, but not the increase that has been observed in the carotenoid content (Figure 1E,F). Thus, Naumann et al. [80] in *Lemna minor* and Mazur et al. [44] in *Sinapis alba* find Tl to induce decreases in both the chlorophyll and the carotenoid content. In *Arundo donax* and *Coix lacryma-jobi*, Pu et al. [48,86] find 50 µg L^−1^ Tl to cause a decrease in the chlorophyll content, while lower concentrations have no effect. The increase in the carotenoid content and the carotenoid/chlorophyll ratio that has been observed in *Dittrichia* could be due to the role of carotenoids as ROS scavengers, thus protecting PSII functionality from oxidative damage [11,98,99,100,101]. The chlorophyll a/chlorophyll b ratio indicates the degree of appression of the thylakoid membranes in the chloroplast, being inversely proportional to the degree of appression, which, in turn, causes the “light harvesting complexes II” (LHCII) to be more closely connected, increasing the light-gathering capacity, and, therefore, the efficiency of energy transmission. In addition, the increase in the thylakoid appression enhances the electron transfer from photosystem II to the cytochrome b_6_f complex, thus increasing non-cyclic photosynthetic electron transport [102]. That ratio is lower in control plants than in those that were grown in the presence of Tl, which would mean that the control plants capture light more efficiently. The interaction between Tl and the -SH groups affects the functionality of the enzyme systems that are involved in the biosynthesis and/or degradation of chlorophylls and the stability of the chloroplasts, as described for Sb toxicity [11,102].

The photosynthetic efficiency of control plants and those that were treated with 10 µM Tl are very close to the values that are described as normal by Murchie and Lawson [103]. The higher concentration Tl treatments show greater decreases, indicating an impairment of the photochemical activity. The stress that is induced by Tl toxicity causes an increase in the minimum fluorescence value (F_0_) (Figure 1G), reflecting an alteration in the photosynthetic apparatus. This reveals that there is excess O_2_^.−^ production in the chloroplasts, which negatively affects the PSII activity. The F_0_ fluorescence originates from the chlorophylls that are associated with the antenna complexes. Its increase thus implies a reduced energy transfer from LHCII to PSII, i.e., there is a disconnection of the antennas in response to stress [104,105]. The F_v_/F_m_ ratio represents the maximum PSII quantum yield, which correlates with the net photosynthesis quantum yield. It is indicative of the level of photoinhibition affecting the PSII complexes [106]. Tl toxicity reduces F_v_/F_m_ (Figure 1H) to different degrees depending on the Tl concentration, these results being similar to those that have been described in other plants [44,47,48,86]. Changes in the PSII activity reduce photosynthesis levels [105,107]. In addition, the ratio F_v_/F_0_, which indicates the maximum ratio of the quantum yields of the photochemical and non-photochemical PSII processes, allows the photosynthesis capacity to be estimated. *Dittrichia viscosa* under Tl toxicity conditions show large decreases in this ratio (Figure 1H), which may indicate an interruption of donor-side PSII photosynthesis [108]. These results indicate that Tl toxicity leads to a strong alteration of photosynthetic activity, affecting PSII, as indicated by the increases in F_0_ and the decreases in the F_v_/F_m_ and F_v_/F_0_ ratios. Recently, Chang et al. [45] have described the negative effect of Tl on the photosynthetic process by decreasing the expression levels of the LCHII subunit genes, thus reducing the aggregation of the LCHII complexes, and, with them, the levels of photosynthesis.

With regard to ROS, by accumulating the Tl preferentially in the roots (Table 2), there is an increase in ROS production, which can cause the lipid peroxidation that is observed in the roots (Figure 2A). This behavior is similar to that described in *Dittrichia* plants in response to stress induced by Sb toxicity [11]. The increase in lipid peroxidation has also been described in response to Tl-induced stress [49,81]. On the contrary, Sirova and Vaculik [109] describe an increase in lipid peroxidation in leaves due to Cd toxicity, but not in roots, since a large part of the Cd is translocated to the leaves, provoking oxidative stress that would damage the membranes. Yao et al. [55] describe a decrease in lipid peroxidation in rice roots that were subjected to Tl(I) toxicity. In *Dittrichia*, TF_Tl_ decreases progressively with increasing Tl (Table 2), and this, together with the increase in SOD activity, may prevent increases in lipid peroxidation in leaves [110]. In the roots, the accumulation of Tl is very high, inducing strong increases in the production of O_2_^.−^ and H_2_O_2_, and, although the SOD activity increases, damage by lipid peroxidation occurs (Figure 2). The stress induced in *Dittrichia* by Tl leads to increases in the content of O_2_^.−^ and H_2_O_2_ and in the SOD activity (Figure 2B–D), which is a response that is similar to that described in other plants [49,81]. On the contrary, while decreases in O_2_^.−^ and H_2_O_2_ content have also been observed, the SOD activity increases [55]. However, the toxicity that is induced by Cd and As induces increases in the production of O_2_^.−^ and H_2_O_2_, as well as in SOD activity [9,23,85,111]. NO and H_2_S perform signaling functions, both under physiological conditions and under stress conditions, intervening at the level of nitration, nitrosation, and persulfidation [112,113]. Tl induces increases in the NO content in *Dittrichia* (Figure 2E), similar to those that have been described in response to toxicity by heavy metals such as As and Sb [10,11,16,114]. However, a decrease in the NO content in As toxicity has also been described [85,115,116]. These contradictory results could be explained by the magnitude of the stress, the plant age, the organ, or the treatment duration [116,117]. Increases in the H_2_S content (Figure 2F) have also been described in response to toxicity by Sb [11], Cd [9,85], and Cr [118]. The amount of H_2_S is greater in leaves than in roots, possibly because of the greater production of H_2_S in chloroplasts than in cytoplasm [119].

AsA and GSH are involved in the activation of the antioxidant systems of plants against stress [120]. In addition, they maintain cellular redox homeostasis [121]. Our results show that Tl toxicity induces alterations in APX, DHAR, MDHAR, and GR activities (Figure 3), as well as increases in AsA content and decreases in DHA, GSH, and GSSG (Figure 4). The AsA content in the roots increases, and that of DHA decreases, thus raising the AsA/DHA ratio (Figure 4A–D). The increase in AsA may be due to the increase in DHAR and GR and a decrease in the APX activity (Figure 3A,B,D), with an increase in H_2_O_2_ content. However, the MDHAR activity decreases (Figure 3C) despite it being the main enzyme responsible for AsA levels. In roots, although there is an increase in NO, the H_2_S levels only increase significantly with 10 µM of Tl, whereas the greater concentrations barely raise this content. In leaves, as in roots, the AsA content increases and the DHA content decreases, with consequent increases in the AsA/DHA ratio. Both DHAR and MDHAR increase, which raises the AsA content, since APX, although increasing in activity, does so to a lesser extent. The increases that are observed in the levels of NO and H_2_S in leaves may increase the APX activity, which is modulated by both S-nitrosation and by persulfidation [122,123]. Espinosa-Vellarino et al. [11] describe Sb toxicity in *Dittrichia* leading to increased AsA and decreased DHA in leaves, with a decrease in both in the roots. On the contrary, decreases in AsA content have been described due to the toxicity of Tl [74], Cd, and As [85,124]. Hasanuzzaman et al. [125], also with Cd toxicity, describe decreases in AsA and increases in DHA.

In the roots, the GSH content does not change with 10 or 50 µM of Tl, but increases with 100 µM Tl, while that of GSSG decreases with 10 and 50 µM and remains at the control levels with 100 µM of Tl. The GSH/GSSG ratio increases with Tl (Figure 4H), and, although there is greater GR activity, the GSH content does not increase in absolute terms, although it does so relative to GSSG. The increased DHAR might consume the GSH that is produced by the GR, or this GSH might be used for other reactions, such as the direct elimination of ROS, phytochelatin synthesis, or direct binding to Tl, which has a high affinity for -SH groups [27,34]. In leaves, GSH and GSSG decrease, as does the GSH/GSSG ratio, which could be due to increased DHAR activity and decreased GR activity. These alterations are similar to those that are described by Hasanuzzaman et al. [125], with a decrease in the content of GSH and redox pool, an increase in APX activity, decreases in DHAR and MDHAR, and variable behavior of the GR. In *Dittrichia* under Sb toxicity [11], there are similar alterations in the leaves, although in the roots, the GSH content also decreases, as well as that of GSSG. The decrease in the GSH content in the leaves contrasts with the increase that has been described in response to Zn toxicity [126]. Alterations in the AsA/DHA and GSH/GSSG ratios are fundamental as a protective mechanism of the cell against ROS [127]. In our case, Tl produces an increase in the AsA/DHA ratio (Figure 4D) in both the roots and the leaves, and in the GSH/GSSG ratio (Figure 4H) in the roots, but with this ratio remaining constant, or slightly lowered, in the leaves. Tl induces an alteration of the cellular redox state. These results show the great capacity of *Dittrichia* to activate antioxidant defense systems, from amino acids, carotenoids, antioxidant enzymes, and AsA, maintaining a reduced state of the main antioxidants, especially in the leaves, in response to Tl stress.

## 5. Conclusions

In *Dittrichia*, the stress induced by Tl led to a major reduction in plant biomass, with reductions in the size and FW, altered the DW/FW ratio, but hardly affected RWC, strongly increased the amount of soluble amino acids, and decreased that of proteins. At the level of nutrient absorption and accumulation, it induced a major decrease in K, Mg, and Fe contents in both the roots and the leaves. On the contrary, Ca, Mn, and Zn accumulated to a greater extent in both of the organs. While chlorophylls decreased, carotenoids increased, possibly to act as a protection system by reducing the photosynthetic efficiency. With regard to stress markers, an increase in lipid peroxidation was observed, especially in the roots. The production of O_2_^.−^, H_2_O_2_, and NO was greater in the roots than in the leaves, increasing in both of the organs in response to Tl toxicity, except for O_2_^.−^ production in roots, which presented fluctuations. There was also greater H_2_S production, especially in the leaves. The activities of SOD, APX, DHAR, MDHAR, and GR increased, except for APX in the roots and GR in the leaves. The components of the AsA–GSH cycle were also affected, with AsA increasing while DHA, GSH, and GSSG decreased. These Tl toxicity-induced alterations modified the AsA/DHA and GSH/GSSG redox status. The effects of Tl could be related to its high -SH group binding affinity, altering the functionality of proteins and the cellular redox state.

## Figures and Tables

**Table 1:** Effect of Tl on the length, fresh weight (FW), dry weight (DW), and fresh weight percentage (%DW) of the *D. viscosa* plants. The data are the means ± SE from ten independent experiments ± SE, each carried out in triplicate. The different letters indicate significant differences at *p* < 0.05.

Treatments	Length (cm)		FW (g)		DW (g)		% DW	
	Roots	Shoots	Roots	Shoots	Roots	Shoots	Roots	Shoots
Control	46.29 ± 2.33a	17.21 ± 1.89a	2.135 ± 0.332a	4.603 ± 0.495a	0.181 ± 0.010a	0.690 ± 0.047a	6.47 ± 0.15b	11.78 ± 0.29c
10 µM Tl	18.10 ± 2.08b	6.55 ± 0.81b	0.532 ± 0.077b	1.267 ± 0.132b	0.079 ± 0.004b	0.237 ± 0.032b	10.05 ± 0.38a	18.03 ± 0.50b
50 µM Tl	19.90 ± 1.32b	6.27 ± 0.50b	0.520 ± 0.075b	1.094 ± 0.113c	0.072 ± 0.008b	0.230 ± 0.031b	9.81 ± 0.73a	17.62 ± 0.87b
100 µM Tl	19.25 ± 1.68b	5.67 ± 0.43b	0.500 ± 0.053b	0.845 ± 0.055d	0.046 ± 0.009c	0.183 ± 0.036c	7.20 ± 0.97b	22.16 ± 1.07a

**Table 2:** Tl content in roots and leaves, bioaccumulation factor (BF), and translocation factor (TF) in *D. viscosa* plants. The data are the means ± SE from 10 independent experiments, each carried out in triplicate. The different letters indicate significant differences at *p* < 0.05. nd = not detect.

Treatments	Tl (mg kg^−1^ DW)		BF		TF
	Roots	Shoots	Roots	Shoots	
Control	1.9d	nd	nd	nd	nd
10 µM Tl	2286.0 ± 378.2c	374.7 ± 48.9b	571a	93a	0.163a
50 µM Tl	7991.2 ± 786.0b	623.9 ± 89.5a	399b	31b	0.078b
100 µM Tl	14,035.7 ± 634.9a	601.1 ± 121.0a	351b	15c	0.043c

**Table 3:** Effect of Tl on the K, Mg, Ca, Fe, Mn, Cu, and Zn contents in roots and shoots of *D. viscosa* plants. The data are the means ± SE from ten independent experiments, each carried out in triplicate. The different letters indicate significant differences at *p* < 0.05.

**Treatments**	**K (mg kg^−1^ DW)**		**Mg (mg kg^−1^ DW)**		**Ca (mg kg^−1^ DW)**			
	**Roots**	**Shoots**	**Roots**	**Shoots**	**Roots**		**Shoots**	
Control	84,014.1 ± 27,699.2a	96,366.0 ± 31,295.4a	34,884.6 ± 2213.3a	6093.3 ± 1696.1a	44,796.5 ± 10,631.2ab		14,465.0 ± 3990.8a	
10 µM Tl	75,916.4 ± 29,047.9a	61,058.8 ± 17,123.4a	11,205.2 ± 4113.9b	4863.9 ± 782.5b	21,418.2 ± 5794.7c		15,267.4 ± 3134.7a	
50 µM Tl	58,217.0 ± 20,738.0b	61,336.0 ± 21,882.6a	12,748.7 ± 5783.5b	4729.2 ± 918.6b	35,963.2 ± 10626.0b		14,249.8 ± 2799.5a	
100 µM Tl	50,594.4 ± 21,353.5b	72,777.5 ± 32,277.6a	13,252.0 ± 4551.0b	5813.6 ± 1731.1a	57413.1 ± 10,935.5a		16,019.9 ± 5930.9a	
**Treatments**	**Fe (mg kg^−1^ DW)**		**Mn (mg kg^−1^ DW)**		**Cu (mg kg^−1^ DW)**		**Zn (mg kg^−1^ DW)**	
	**Roots**	**Shoots**	**Roots**	**Shoots**	**Roots**	**Shoots**	**Roots**	**Shoots**
Control	5466.5 ± 3559.0a	134.9 ± 68.2a	882.2 ± 131.3c	55.3 ± 5.0c	45.7 ± 12.5	12.1 ± 2.8a	52.7 ± 20.7b	38.7 ± 7.8
10 µM Tl	2530.1 ± 1131.4b	151.2 ± 75.8a	1450.3 ± 694.1a	225.9 ± 93.9a	41.9 ± 12.5	8.4 ± 2.6b	83.2 ± 35.8ab	51.2 ± 17.9a
50 µM Tl	3031.6 ± 1149.0b	111.6 ± 69.0b	1083.1 ± 128.5b	144.6 ± 69.8b	51.2 ± 17.3	8.3 ± 4.0b	103.0 ± 32.6a	47.0 ± 23.9ab
100 µM Tl	4134.8 ± 1034.3a	84.1 ± 39.5b	1345.4 ± 17.3a	172.6 ± 72.3a	74.4 ± 15.5a	8.3 ± 2.6b	161.7 ± 56.5a	53.8 ± 23.4a

**Table 4:** Effect of Tl on the soluble amino acid and protein contents in *D. viscosa* plants. The data are the means ± SE from ten independent experiments, each carried out in triplicate. The different letters indicate significant differences at *p* < 0.05.

Treatments	Total Soluble Amino Acids (mg g^−1^ FW)		Total Proteins (mg g^−1^ FW)	
	Roots	Leaves	Roots	Leaves
Control	2.375 ± 0.517b	2.540 ± 0.236b	2.12 ± 0.18a	2.82 ± 0.43a
10 µM Tl	4.176 ± 0.461a	10.816 ± 1.628a	2.39 ± 0.07a	1.60 ± 0.17c
50 µM Tl	4.439 ± 0.665a	10.171 ± 0.920a	1.77 ± 0.16b	1.90 ± 0.12b
100 µM Tl	4.459 ± 0.759a	11.192 ± 1.015a	1.25 ± 0.28c	1.82 ± 0.21b

## Data Availability

The data presented in this article will be made available without any reservation.

## Supplementary Materials

The following are available online at https://www.mdpi.com/article/10.3390/antiox12030678/s1, Figure S1: *Dittrichia viscosa* plants subjected to Tl toxicity for 7 days; Table S1: Effect of Tl toxicity on the relative water content (RWC) in roots and leaves of *D. viscosa* plants; Table S2: Effect of Tl toxicity on the translocation factor of K (TF_K_), Mg (TF_Mg_), Ca (TF_Ca_), Fe (TF_Fe_), Mn (TF_Mn_), Cu (TF_Cu_), and (TF_Zn_) of *D. viscosa* plants.
