# Supplementary material for: Effect of Thallium(I) on Growth, Nutrient Absorption, Photosynthetic Pigments, and Antioxidant Response of Dittrichia Plants
Source: Antioxidants (Basel). 2023 Mar 9;12(3):678. doi: 10.3390/antiox12030678 (PMC10045270; doi:10.3390/antiox12030678)
Supplement: Supplementary file 1 [file antioxidants-12-00678-s001.zip › antioxidants-2218418-supplementary.pdf]

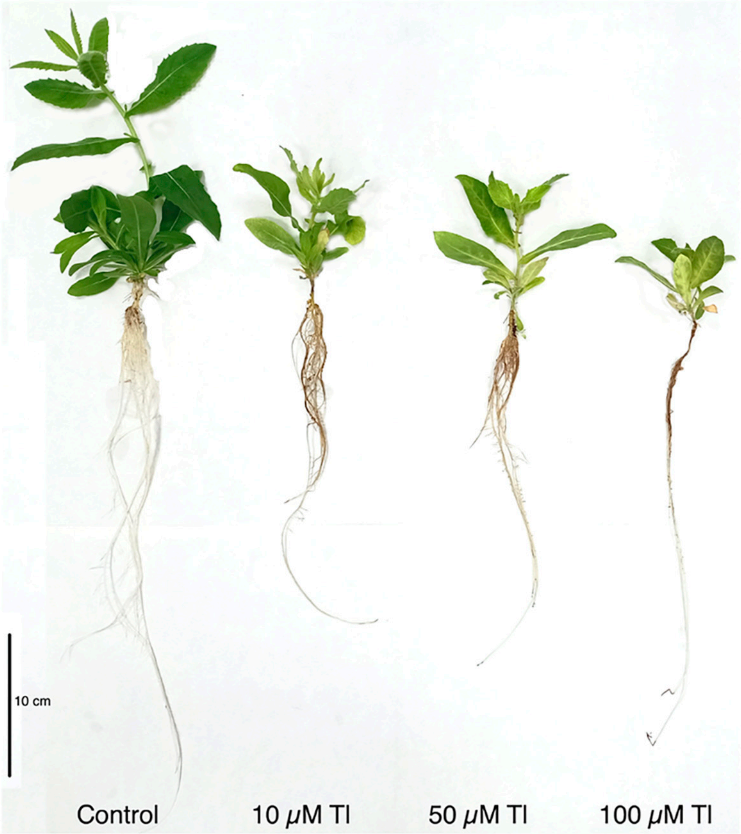

Figure S1. *Dittrichia viscosa* plants subjected to Tl toxicity for 7 days.

Table S1. Effect of Tl toxicity on the relative water content (RWC) in roots and leaves of *D. viscosa* plants. The data are the means ± SE from ten independent experiments.

Treatments	Roots	Leaves
Control	95.00±1,12a	91.65±1.95a
10 μM Tl	94,71±0.97a	91.70±1.79a
50 μM Tl	97.02±1.58a	88.75±0.57ab
100 μM Tl	96.53±0.81a	84.75±0.97a

The different letters indicate significant differences at $p < 0.05$.

Table S2. Effect of Tl toxicity on the translocation factor of K (TF_K), Mg (TF_{Mg}), Ca (TF_{Ca}), Fe (TF_{Fe}), Mn (TF_{Mn}), Cu (TF_{Cu}), and (TF_{Zn}) of *D. viscosa* plants. The data are the means ± SE from ten independent experiments.

Treatments	TF_K	TF_{Mg}	TF_{Ca}	TF_{Fe}	TF_{Mn}	TF_{Cu}	TF_{Zn}
Control	1.15±0.01b	0.24±0.10b	0.32±0.01b	0.03±0.01b	0.07±0.01b	0.27±0.01a	0.80±0.16a
10 μM Tl	0.84±0.05c	0.47±0.10a	0.73±0.05a	0.06±0.00a	0.16±0.01a	0.20±0.01b	0.64±0.06b
50 μM Tl	1.05±0.00b	0.43±0.12a	0.41±0.04b	0.03±0.01b	0.13±0.04a	0.15±0.02c	0.42±0.09c
100 μM Tl	1.42±0.03a	0.45±0.02a	0.27±0.05c	0.02±0.01c	0.13±0.05a	0.11±0.01d	0.32±0.03d

The different letters indicate significant differences at $p < 0.05$.
